# Attention to the principles of exercise training in exercise studies on prostate cancer survivors: a systematic review

**DOI:** 10.1186/s12885-019-5520-9

**Published:** 2019-04-05

**Authors:** Sarah E. Neil-Sztramko, Mary E. Medysky, Kristin L. Campbell, Kelcey A. Bland, Kerri M. Winters-Stone

**Affiliations:** 1 School of Nursing, McMaster University, 175 Longwood Road S, Suite 210a, Hamilton, ON L8P 0A1 Canada; 2 School of Nursing, Oregon Health and Science University, Portland, Oregon, USA; 3 Department of Physical Therapy, University of British Columbia, Vancouver, British Columbia Canada; 4 Mary MacKillop Institute for Health Research, Australian Catholic University, Melbourne, Victoria Australia; 5 Knight Cancer Institute, Oregon Health and Science University, Portland, Oregon, USA

**Keywords:** Prostate cancer, Exercise, Systematic review, Exercise prescription

## Abstract

**Background:**

The purpose of this review is to update previously published reviews on exercise programming in exercise trials in prostate cancer survivors. We evaluated: 1) the application of the principles of exercise training in prescribed programs; 2) the reporting of the components of the exercise prescription; and 3) the reporting of adherence of participants to the prescribed programs.

**Methods:**

Building upon a previous review, a systematic review was conducted searching OVID Medline, Embase, CINAHL, and SPORTDiscus databases from 2012-2017. Randomized controlled trials of at least four weeks of aerobic and/or resistance exercise in men diagnosed with prostate cancer that reported physical fitness outcomes, including body composition were eligible for inclusion.

**Results:**

Specificity was appropriately applied by 93%, progression by 55%, overload by 48%, initial values by 55%, and diminishing returns by 28% of eligible studies. No study adequately applied the principle of reversibility. Most (79%) studies reported all components of the exercise prescription in the study methods, but no study reported all components of adherence to the prescribed intervention in the study results.

**Conclusions:**

Application of standard exercise training principles is inadequate in exercise trials in men with prostate cancer and could possibly lead to an inadequate exercise stimulus. While many studies report the basic components of the exercise prescription in their study methods, full reporting of actual exercise completed is needed to advance our understanding of the optimal exercise dose for men with prostate cancer and promote translation of controlled trials to practice.

## Background

Prostate cancer is the most commonly diagnosed cancer in men in developed countries, such as Canada, and is one of the most treatable cancers, with five-year survival rates of 95% [1]. Depending on the stage of disease, treatment options can range from active surveillance to radical prostatectomy, radiation therapy, androgen deprivation therapy, and sometimes chemotherapy [2]. These treatments can have a number of deleterious effects on other health outcomes such as reduced bone mineral density, physical function, and quality of life, along with altered body composition (i.e., gain in fat mass and reduction in lean mass) [3, 4]. These adverse health outcomes are a direct result of cancer and treatment but may also indirectly result from a decline in physical activity that can occur during treatment [5].

Previous systematic reviews have summarized the existing evidence for the role of exercise in improving physical fitness (i.e., strength and aerobic fitness), fatigue and lean body mass [4, 6], with resistance training appearing to be particularly beneficial in counteracting adverse changes in body composition [7]. Observational studies have also suggested an important role for physical activity in reducing mortality after a prostate cancer diagnosis [8]. Despite this evidence, there remains uncertainty around the most efficacious exercise prescriptions for improving specific outcomes across this heterogeneous population. This knowledge gap limits the translation of prescriptions from tightly controlled efficacy studies in a research setting to practice in the broader population of prostate cancer survivors who likely vary in their exercise capacity and may exercise in less supervised settings.

In 2012 and 2014, our team published a pair of systematic reviews to evaluate the application of the standard principles of exercise training and to summarize reporting of and adherence to an exercise prescription in studies of breast cancer survivors [9] and in survivors of cancers other than breast [10]. These reviews were prompted by our observation that well-established exercise training principles from the field of exercise physiology (Table 1 [11]) were either not considered in the design of exercise oncology trials, or were misapplied. In our reviews, and in a recently published update of our first breast cancer review [12], across 113 trials, none applied all of the principles of exercise training and only two [13, 14] reported all of the components of the exercise prescription in their methods and results.

**Table 1:** Exercise training principles

Principle	Criteria for this review	Example
**Specificity:** Training adaptations are specific to the organ system or muscles trained with exercise	Appropriate population targeted and modality selected based on primary outcome	Aerobic exercise such as brisk walking is more appropriate for an intervention aimed at increasing cardiovascular fitness than strength training
**Progression:** Over time, the body adapts to exercise. For continued improvement, the volume or intensity of training must be increased	Stated exercise programme was progressive and outlined training progression	Increase duration of walking program by 5% every two weeks depending on exercise tolerance
**Overload:** For an intervention to improve fitness, the training volume must exceed current habitual physical activity and/or training levels	Rationale provided that programme was of sufficient intensity/exercise prescribed relative to baseline capacity	Prescribing intensity in a resistance training program based on % of measured and/or estimated 1-repetition maximum
**Initial values:** Improvements in the outcome of interest will be greatest in those with lower initial values	Selected population with low level of primary outcome measure and/or baseline physical activity levels	Selecting a sample with high baseline fatigue levels to participate in an aerobic training program to increase cardiovascular fitness and reduce fatigue
**Reversibility:** Once a training stimulus is removed, fitness levels will eventually return to baseline	Performed follow-up assessment on participants who decreased or stopped exercise training after conclusion of intervention	Participants who maintained training after a supervised exercise program preserved strength whereas those who stopped exercising returned to baseline
**Diminishing returns:** The expected degree of improvement in fitness decreases as individuals become more fit, thereby increasing the effort required for further improvements. Also known as the ‘ceiling effect’	Performed follow-up assessment of primary outcomes on participants who continued to exercise after conclusion of intervention	Gains in muscle strength are greatest in the first half of a training program unless the training stimulus continually increases

Since the publication of the 2014 review of adults with cancer other than breast, a large number of randomized controlled trials (RCTs) in prostate cancer survivors have been published, meaning findings from our last review may now be out of date. Treatments for prostate cancer, such as androgen deprivation therapy, are accompanied by unique side effects, and strong potential for exercise to mitigate these effects. Given that the high survival rate for prostate cancer results in a large population of long term cancer survivors, and the larger number of new trials in men with prostate cancer, a separate review focused exclusively on exercise in prostate cancer survivors could best inform the research field and clinical practice. The purpose of this review is to summarize the published literature on exercise studies conducted in men diagnosed with prostate cancer, with a particularly focus on evaluating 1) the principles of exercise training in the design of the exercise prescription; 2) reporting of the components of the exercise prescription (i.e., frequency, intensity, time and type, or ‘FITT’) in the study methods and 3) adherence of participants to the intervention prescribed in the study results.

## Methods

Using the same protocol as our set of previously published reviews [9, 10], and recently published update in studies of breast cancer survivors [12], Medline, CINAHL, SPORTDiscus and EMBASE databases were searched from January 1, 2012 to August 21, 2017. This search complimented our previous search (completed to December 31, 2011) of exercise studies conducted in survivors of all cancers, other than breast. This review includes the seven papers from the original review [10] that included only men diagnosed with prostate cancer, and any new papers published between 2012 and 2017. The previous subject heading terms related to cancer (cancer, neoplasms, carcinoma) and exercise (exercise, physical activity, aerobic, resistance, walking, etc.) specific to each database were used and combined with the AND term. The search was then limited to English-language publications in peer-reviewed journals. Key publications, including relevant systematic reviews identified during the literature search were hand-searched for relevant trials.

Included studies were conducted exclusively in men diagnosed with prostate cancer, regardless of treatment type or stage of treatment. Eligible studies were required to be RCTs with one or more treatment arms involving at least four weeks of aerobic and/or resistance exercise. Alternative forms of exercise, such as yoga, Pilates, or Tai Chi, as well as therapeutic interventions (i.e., physical therapy, stretching) were excluded. Studies that focused primarily on physical activity behaviour change and those that only reported levels of physical activity or psychosocial outcomes were excluded. In line with our previous criteria, all studies were required to report at least one relevant physiological outcome related to exercise (e.g., aerobic capacity, muscular strength, physical function, or body composition). Secondary publications from previously included trials were added to the database of included articles to allow for review of previously extracted data and updates regarding inclusion of information on use of exercise training principles, exercise prescription or adherence to exercise prescription data but were treated as singular studies.

Two reviewers (SNS and MM) independently determined eligibility using an online software system (Covidence Systematic Review software, Veritas Health Innovation, Melbourne, Australia). Each reviewer first inspected the title and abstract of each study and full-text versions of relevant papers were obtained and further reviewed for eligibility. Discrepancies were resolved by consensus and the input of another member of the study team (KWS) when required.

All relevant data were extracted using the online software system, in duplicate, with discrepancies resolved by consensus. Data extracted included sample size, timing of intervention delivery (during or after treatment), treatment type, intervention delivery mode (supervised or home-based), intervention duration, timing of follow-up measures, primary and secondary outcomes, and study findings. The exercise prescription was abstracted according to the ‘FITT’ format from each publication’s methods section, including frequency (number of sessions per week), intensity (relative or absolute intensity of exercise), time (duration of exercise) and type of exercise.

For every described exercise prescription, the two reviewers independently assigned a rating for the use of each principle of exercise training (see Table 1). Application of a principle was assigned a ‘+’ when the application was clearly reported and an ‘NR’ (not reported) if there was no indication that the principle was used in the exercise prescription. A ‘?’ was assigned when the principle was mentioned but not described, inconsistently applied, or otherwise unclear. Adequate reporting of the prescription according to the FITT format, and participant adherence to the prescription was also assigned a ‘+’, ‘NR’ or ‘?’. For multi-arm trials comparing different exercise interventions, the application of the principles of exercise training, and the exercise prescription was evaluated separately for each intervention arm. For trials that were previously included, newly identified articles were screened for new information, to determine whether the previously assigned ratings should be altered, but were not counted as another independent trial.

As described previously, the number and percentage of studies that met each criterion for attention to principles of exercise training and reporting of exercise prescribed and completed was calculated and reported. Due to the small number of prostate cancer studies included in the first review, previously included and newly identified studies are presented together in this review. Due to the relatively small number of prostate cancer trials, we were not powered for comparisons of reporting patterns between studies in our first review (< 2012) and new studies identified in this review (> 2012). Thus, we present descriptive data only.

## Results

**Fig. 1:**
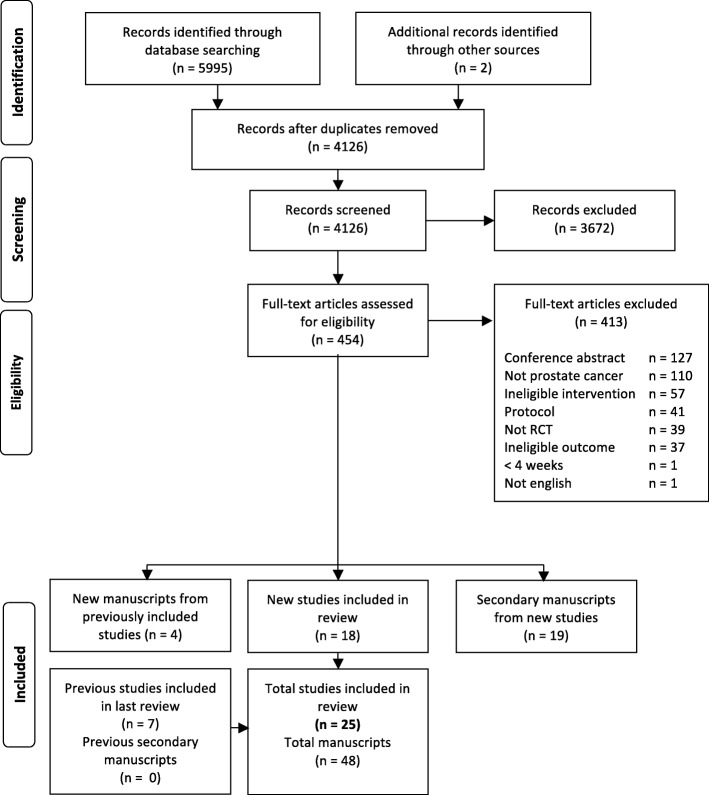
PRISMA diagram of study selection

Study identification, screening, and eligibility information is outlined in the PRISMA diagram (Fig. 1). In total, 41 papers were identified from our search and included here. Of these, there were 37 papers from 18 unique trials published after 2012, and four secondary papers [15–18] from the seven trials included in our previous review [19–25], for a total of 25 included trials. Of the four new papers from previously included studies two reported on different outcome measures [16, 18] and the other two reported secondary analyses of original outcomes [15, 17]. After reviewing newly published papers from previously included trials, none of their ratings were changed from our previous review.

Across included trials, seven (28%) prescribed aerobic exercise only [22, 25–30], five (20%) prescribed resistance exercise only [23, 31–34], ten (40%) prescribed combined aerobic + resistance exercise [19–21, 35–41] and three (12%) were multi-arm trials (seven intervention arms) comparing aerobic to resistance or aerobic + resistance exercise [24, 42, 43] (Table 2). The interventions themselves ranged from four weeks to two years in duration and consisted of either entirely supervised (*n* = 10, 40%) [21–24, 30, 32, 34, 35, 38, 39], entirely home-based (*n* = 5, 20%) [25–27, 40, 41] or a combination of supervised + home-based exercise (*n* = 10, 40%) [19, 20, 28, 29, 31, 33, 36, 37, 42, 43].

**Table 2:** Description of included studies

Authors, Year	Timing	Treatment	N	Intervention	Length (weeks)	Follow-up(weeks)	Primary Outcome* (Tool)	Other Outcomes (Tool)
Aerobic Exercise Only								
Eriksen et al, 2017 [26]	During	AS	26	Home	26	12, 37, 52	NR	VO_2 peak_ (max cycle), lipids, insulin, glucose, PSA, FFM, FM, BMI, WC
Hvid et al, 2016 [27]	Mixed	AS or RP	25	Home	104	12, 24, 36, 52, 78	NR	VO_2 peak_ (max cycle), BC (DXA), HOMA-IR (OGTT), TC, LDL, HDL, TG, TNF-α, IL-6, adiponectin, leptin, IGF-1, IGFBP-1, glucose, PSA
Jones et al, 2014 [28]	Mixed	RP	50	Sup + Home	26	12, 52	Erectile Function (IIEF)	VO_2 peak_ (max TM), peripheral artery FMD, glucose, TC, TG, BC (BodPod)
Monga et al, 2007 [22]	During	RT	21	Sup	8	–	Fatigue (PFS)	VO_2 peak_ (submax TM), strength (sit-stand)
Pernar et al, 2017 [29]	After	Mixed	41	Sup + Home	11	–	NR	BW, WC, BP, CRP, C-peptide, HDL, LDL, testosterone, SHBG
Uth et al, 2014 [30]	During	ADT	57	Sup	12	32	LBM (DXA)	BMD, BMC, FM (DXA), BW, BMI, WC, HC, WHR, VO_2 peak_ (submax TM), sit-stand, counter-movement jump, stair climb, strength (1RM, knee ext), bone markers
Windsor et al, 2004 [25]	During	RT	66	Home	4	–	Fatigue (BFI), Aer fitness (Shuttle walk)	NR
Resistance Exercise Only								
Nilsen et al, 2015 [31]	During	ADT	58	Sup + Home	16	–	NR	LBM, BMD, FM, %BF, BW, BMI (DXA), 1RM (knee ext., leg, chest, shoulder press), sit-stand, stair climb, aer fitness (shuttle walk); muscle cell markers, fiber
Norris et al, 2015 [32]	Mixed	Mixed	30	Sup (High)	12	–	Strength (est 1RM, bench press, leg press)	Physical function (6MWT, 8 ft. TUG, 30s chair stands, 30s arm curls, S&R, back scratch); BW, BMI,
				Sup (Low)				
Segal et al, 2003 [23]	During	ADT	155	Sup	12	–	Fatigue (FACT-F)	Muscle endurance (Standard Load), BW, BMI, WC (SkF)
Winters-Stone et al, 2014 [33]	During	ADT	51	Sup + Home	52	–	BMD (DXA)	Bone turnover, LM, FM (DXA), insulin, IGF-1, SHBG, testosterone, strength (1RM, leg and chest press), physical function (5 chair stand time, 4 m walk speed)
Winters-Stone et al, 2016 [34]	After	RT ± CT	64	Sup	24	12	Function (SPPB), MM, FM, %BF (DXA), fatigue (PFS), QoL (SF-36), strength (1RM leg, bench press)	NR
Aerobic + Resistance Exercise								
Bourke et al, 2011 [19]	During	ADT	100	Sup + Home	12	6, 24	QoL (FACT-P), DBP	VO_2 peak_ (submax TM), strength (Iso-dyn, subgroup), BMI, IGF-1, IGFBP-1, IGFBP-3, insulin, PSA, androgen, testosterone, SHBG; Subgroup: Brachial artery FMD, GTN-mediated brachial artery dilation
Cormie et al, 2015 [35]	During	ADT	63	Sup	12	–	LM, FM (DXA)	BMD (DXA), aer fitness (400 m walk), strength (1RM leg press, chest press, seated row), chair stand, stair climb, BP, CRP, TC, TG, insulin, glucose, HbA1C, bone turnover, vitamin D, testosterone, PSA
Culos-Reed et al, 2010 [20]	During	ADT	100	Sup + Home	16	8, 26	SR-PA (Godin)	Aer fitness (6MWT), strength (Iso-dyn), S&R, BP, HR
Galvao et al, 2010 [21]	During	ADT	57	Sup	12	–	LBM, FM, %BF (DXA)	Strength (1RM), Endurance (Time at 70% 1RM), physical function (sit-stand, 6 & 400 m walk), testosterone, PSA, insulin, glucose, lipids, CRP
Galvao et al, 2014 [36]	After	ADT + RT	100	Sup + Home	52	26	Aer fitness (400 m walk)	Physical function (chair stands), WC, testosterone, PSA, insulin, TC, TG, LDL, HDL, HbA1C, glucose, BP Subgroup: Strength (1 RM, chest and leg press), FM, %BF, adiposity (DXA)
Gaskin et al, 2017 [37]	After	Mixed	320	Sup + Home	12	26, 52	SR-PA (LTEQ)	RHR, BP, aer fitness (6MWT), strength (1RM), physical function (30s sit-stand), BW, BMI, WC, HC, upper arm, chest, thigh circumference
Hojan et al, 2016 [38]	During	RT + ADT	55	Sup	8	–	NR	PSA, Hb, WBC, RBC, neutrophil, lymphocytes, platelets, monocytes, IL-B, IL-6, TNF-α, Aer fitness (6MWT)
Hojan et al, 2017 [39]	During	RT + ADT	72	Sup	52	8	Aer fitness (6MWT), IL-1B, IL-6, TNF-a, BMI, WHR, WC, TC, HDL, LDL, TG, AST, ALT	NR
Kim, 2018 [40]	During	ADT	51	Home	24	–	BMD (DXA), Bone turnover markers (bs-ALP, NTx)	Strength (grip, hip, HHD), 30s chair-stand, TUG
Sajid et al, 2016 [41]	During	ADT	19	Home	6	12	Function (SPPB)	Aer fitness (6MWT), strength (grip, chest press reps), FM, LM, MM (DXA)
				Technology				
Aerobic or Resistance Exercise (Multi-Arm Trials)								
Santa Mina et al, 2013 [42]	During	ADT	26	Sup + Home Aer	24	12, 52	QoL (FACT-P, Patient-Oriented Prostate Utility Scale), Fatigue (FACT-F)	VO_2 peak_ (Submax TM), grip strength, BW, BMI, %BF, WC, IGF-1, IGFBP-3, leptin, adiponectin
				Sup + Home Res				
Segal et al, 2009 [24]	During	RT	121	Sup Aer	24	–	Fatigue (FACT-F)	VO_2 peak_ (max TM), strength (8RM), BW, BC (DXA), testosterone, PSA, Hb, lipids
				Sup Res				
Wall et al, 2017 [43]	During	ADT	163	Sup + Home Aer + Res	52 (26)	–	BMD (DXA), LM, FM, Trunk fat, % BF, (DXA), VO_2 peak_ (Max TM)	RMR, BP, arterial stiffness, HbA1C, testosterone, insulin, PSA, TG, LDL, HDL, TC, glucose, CRP, bs-ALP, PINP, BW, strength (1-RM), endurance (#reps @ 70% 1RM, chest & leg press), chair stands, stair climb, 6 m backward walk, 400 m walk time
				Sup + Home Impact + Res				
				Home Aer	26	–		

Legend: 6MWT: 6 min walk test; ADT: androgen deprivation therapy; Aer: aerobic; ALT: alanine transaminase; AS: active surveillance; AST: aspartate transaminase; BC: body composition; BFI: brief fatigue inventory; BMC: bone mineral content; BMD: bone mineral density; BMI: body mass index; BP: blood pressure; bs-ALP: bone specific alkaline phosphatase; BW: body weight; CRP: c-reactive protein; CT: chemotherapy; DBP: diastolic blood pressure; DXA: dual energy x-ray absorptiometry; ext: extension; FACT-F: functional assessment of cancer therapy – fatigue; FACT-P: functional assessment of cancer therapy – prostate; FFM: fat free mass; FM: fat mass; FMD: flow mediated dilation; Hb: hemoglobin; HbA1C: glycosylated hemoglobin; HC: hip circumference; HDL: high density lipoprotein; HHD: hand held dynamometry; HOMA-IR: homeostatic assessment of insulin resistance; IGF: insulin-like growth factor; IGFBP: insulin-like growth factor binding protein; IIEF: international index of erectile function; IL: interleukin; Iso-dyn: isometric dynamometry; LBM: lean body mass; LDL: low density lipoprotein; LM: lean mass; LTEQ: leisure time exercise questionnaire; Max: maximum; MM: muscle mass; NR: not reported; NTx: type 1 cross-linked N-telopeptide; OGTT: oral glucose tolerance test; PFS: piper fatigue scale; PINP: Pro collagen Type 1 N-Terminal Propeptide; PSA: prostate specific antigen; QoL: quality of life; RBC: red blood cell; Res: resistance; RHR: resting heart rate; RM: repetition maximum; RMR: resting metabolic rate; RP: radical prostatectomy; Res: resistance training; RT: radiation therapy; S&R: sit and reach; SHBG: sex hormone binding globulin; SkF: skin fold; SPPB: short physical performance battery; SR-PA: self-reported physical activity; submax: submaximal; Sup: supervised; TC: total cholesterol; TG: triglycerides; TM: treadmill; TNF-α: tumour necrosis factor-alpha; TUG: timed up and go; VO_2__peak_: peak oxygen consumption; WBC: white blood cell; WC: waist circumference; WHR: waist-hip ratio; *Where specifically stated

### Application of the principles of exercise training

**Fig. 2:**
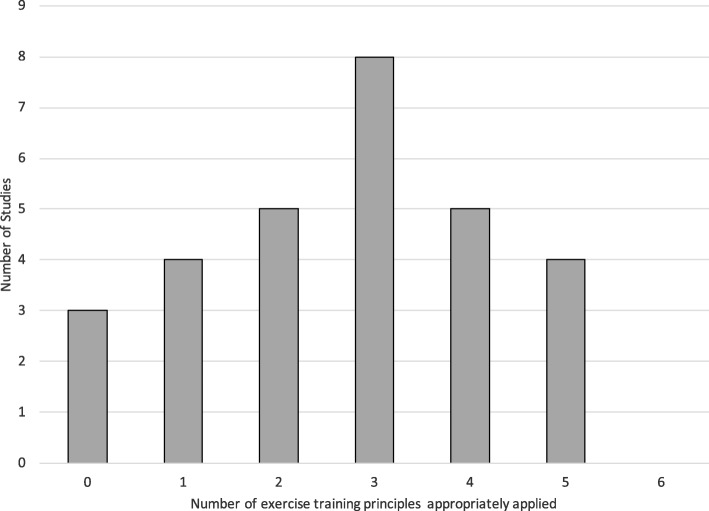
Number of exercise training principles applied across included trials

Ratings of the principles of exercise training for all included trials are displayed in Table 3. No included trial reported attention to all six evaluated principles of exercise training. Only nine (33%) trials appropriately applied more than half of the principles (i.e., four or five of a possible six), eight (28%) trials applied half of the principles, and twelve (41%) trials applied fewer than half other principles (Fig. 2).

**Table 3:** Application of the principles of exercise training and outcomes

Authors, Year	Sp	Pr	OV	IV	Rev	DR	Significant results
Aerobic Exercise Only							
Eriksen et al, 2017 [26]	NR	NR	NR	NR	?	?	↑VO_2 peak_ (6mo only)
Hvid et al, 2016 [27]	?	+	+	+	?	?	6 mo: ↑VO_2 peak_, adiponectin, IGFBP-1; ↓FM, trunk mass, gynoid FM, android fat; 24 mo: ↓ FM, trunk mass, gynoid FM, android fat, TG, IGF-1, glucose
Jones et al, 2014 [28]	+	+	+	+	?	?	↑FMD, VO_2 peak_
Monga et al, 2007 [22]	+	?	+	NR	NR	NR	↑ VO_2 peak_, Strength; ↓ Fatigue*
Pernar et al, 2017 [29]	NR	NR	NR	NR	NR	NR	↑ HDL
Uth et al, 2014 [30]	NR	+	NR	+	?	?	EoS: ↑LBM*, strength, BMC, P1NP, osteocalcin; 32 w: ↑BMD, counter jump, stair climb
Windsor et al, 2004 [25]	+	NR	?	NR	?	?	↑ Aer fitness*
Resistance Exercise Only							
Nilsen et al, 2015 [31]	+	+	+	+	NR	NR	↑Muscle fiber cross-sectional area, strength (leg ext., leg, chest, shoulder press), sit-stand; LBM (lower & upper)*
Norris et al, 2015 (High) [32]	+	+	+	+	NR	NR	No between group difference
Norris et al, 2015 (Low) [32]	+	+	+	+	NR	NR	No between group difference
Segal et al, 2003 [23]	+	+	+	NR	NR	NR	↓ Fatigue*; ↑Muscle endurance
Winters-Stone et al, 2014 [33]	+	+	+	+	NR	?	↓ FM, ↑strength
Winters-Stone et al, 2016 [34]	+	+	+	+	NR	+	↑Upper body strength*
Aerobic and Resistance Exercise							
Bourke et al, 2011 [19]	+	+	NR	+	?	?	↑QoL (12w only)*; VO_2 peak_, strength, FMD, skeletal MM, SHBG (subgroup only)
Cormie et al, 2015 [35]	+	+	NR	+	NR	NR	↑Appendicular LM*, Aer fit, strength, chair stands; ↓ FM*, % BF*, TC
Culos-Reed et al, 2010 [20]	+	NR	NR	NR	NR	NR	↑ SR-PA*
Galvao et al, 2010 [21]	+	?	?	NR	NR	NR	↑ LBM*, Strength, Endurance, 6 m walk; ↓ CRP
Galvao et al, 2014 [36]	+	?	NR	+	NR	+	↑ Aer fitness*, chair stands, strength (6 & 12 mo); appendicular MM (6 mo only), HDL; ↓ TC (12 mo only)
Gaskin et al, 2017 [37]	+	?	NR	NR	?	?	↑ Vigorous SR-PA (12wk & 6 mo only)*; Aer fitness, strength, sit-stand; ↓ HC, RHR (12w only)
Hojan et al, 2016 [38]	+	NR	NR	+	NR	NR	↑Aer fitness
Hojan et al, 2017 [39]	+	?	NR	+	NR	+	↓ BW, BMI*, WHR*, PSA, IL-6*, ↑ Aer fitness*
Kim et al, 2018 [40]	+	+	+	+	NR	NR	↑ Grip strength (left hand), 30s chair stands
Sajid et al, 2016 (Home) [41]	?	NR	NR	NR	NR	NR	↑ SPPB (vs. control)*
Sajid et al, 2016 (Tech) [41]	?	NR	NR	NR	NR	NR	None
Aerobic or Resistance Exercise (Multi-Arm Trials)							
Santa Mina et al, 2013 (Aer) [42]	+	NR	NR	NR	?	+	↓ BW, WC, BMI (3 mo only)
Santa Mina et al, 2013 (Res) [42]	+	NR	NR	NR	?	+	↑ IGFBP-3 (6 mo only)
Sega et al, 2009 (Aer) [24]	+	+	+	NR	NR	NR	↑ VO_2 peak_
Segal et al, 2009 (Res) [24]	+	+	+	NR	NR	NR	↑ VO_2 peak_, Strength; No %BF; ↓ TG
Wall et al, 2017 (Aer + Res)	+	+	+	+	NR	+	↑ Strength (6 mo only); Vs. control only: ↑VO_2 peak_, LM*; ↓ glucose, FM*, trunk FM*, % BF*
Wall et al, 2017 (Impact + Res) [43]	+	+	+	+	NR	+	↑ Strength (6, 12 mo)
Wall et al, 2017 (Aer) [43]	+	+	+	+	NR	+	None

Legend: Aer: aerobic; BMC: bone mineral content; BMD: bone mineral density; BMI: body mass index; BW: body weight; CRP: c-reactive protein; DR: diminishing returns; ext: extension; FM: fat mass; FMD: flow mediated dilation; HC: hip circumference; HDL: high density lipoprotein; IGF: insulin-like growth factor; IGFBP: insulin-like growth factor binding protein; IL: interleukin; IV: initial values; LBM: lean body mass; LM: lean mass; MM: muscle mass; mo: months; NR: not reported; OV: overload; Pr: progression; PSA: prostate specific antigen; QoL: quality of life; Res: resistance; Rev: reversibility; RHR: resting heart rate; SHBG: sex hormone binding globulin; Sp: specificity; SPPB: short physical performance battery; SR-PA: self-reported physical activity; TC: total cholesterol; TG: triglycerides; VO_2 peak_: peak oxygen consumption; WC: waist circumference; WHR: waist-hip ratio; * Primary outcome

Specificity was appropriately applied by three (43%) aerobic trials [22, 25, 28], all five (100%) resistance trials [23, 31–34], nine (90%) combined trials [19–21, 35–40] and all seven (100%) multi-arm trials [24, 42, 43]. Specificity was unclear in one (14%) aerobic [27] and one (10%) combined study [41]. Three (43%) aerobic studies did not attend to the principle of specificity [26, 29, 30].

Progression was appropriately reported by three (43%) aerobic [27, 28, 30], all five (100%) resistance [23, 31–34], three (30%) combined [19, 35, 40] and five (71%) multi-arm trials [24, 43]. Progression was unclear in one (14%) aerobic [22], and four (40%) combined trials [21, 36, 37, 39]. The principle of progression was not attended to in three (43%) aerobic [25, 26, 29], three (30%) combined [20, 38, 41] and two (29%) multi-arm trials [42].

Across all studies, three (43%) aerobic [22, 27, 28], all five (100%) resistance [23, 31–34], one (10%) combined [40], and five (71%) multi-arm trials [24, 43] adequately reported use of the principle of overload. One (14%) aerobic [25] and one (10%) combined study [36] were unclear in their application. The remaining three (43%) aerobic [26, 29, 30], eight (80%) combined [19, 20, 35–39, 41] and two (29%) multi-arm trials [42] did not report applying the principle of overload in the development of the exercise intervention.

The principle of initial values was adequately reported within three (43%) aerobic [27, 28, 30], four (80%) resistance [31–34], six (60%) combined [19, 35, 36, 38–40] and three (43%) multi-arm trials [43]. Attention to initial values was not at all reported in the remaining four (57%) aerobic [22, 25, 26, 29], one (20%) resistance [23], four (40%) combined [20, 21, 37, 41] and four (57%) multi-arm trials [24, 42]. No trials were assigned an unclear rating for initial values.

No included trial adequately reported attention to the principle of reversibility. Five (71%) aerobic [25–28, 30], two (20%) combined [19, 37], and two (29%) multi-arm trials [42] were assigned an unclear for this principle. The remaining two (29%) aerobic [22, 29], all five (100%) resistance [23, 31–34], eight (80%) combined [20, 21, 35, 36, 38–41] and five (71%) multi-arm trials [24, 43] did not report reversibility at all.

Finally, diminishing returns was reported by one (20%) resistance training trial [34], two (20%) combined trials [36, 39], and five (71%) multi-arm trials [42, 43]. Diminishing returns was unclear in five (71%) aerobic [25–28, 30], one (20%) resistance [33], and two (20%) combined interventions [19, 37]. It was not at all reported in the remaining two (29%) aerobic [22, 29], three (60%) resistance [23, 31, 32], six (60%) combined [20, 21, 35, 38, 40, 41] and two (29%) multi-arm trials [24].

### Reporting of the components of the exercise prescription

**Fig. 3:**
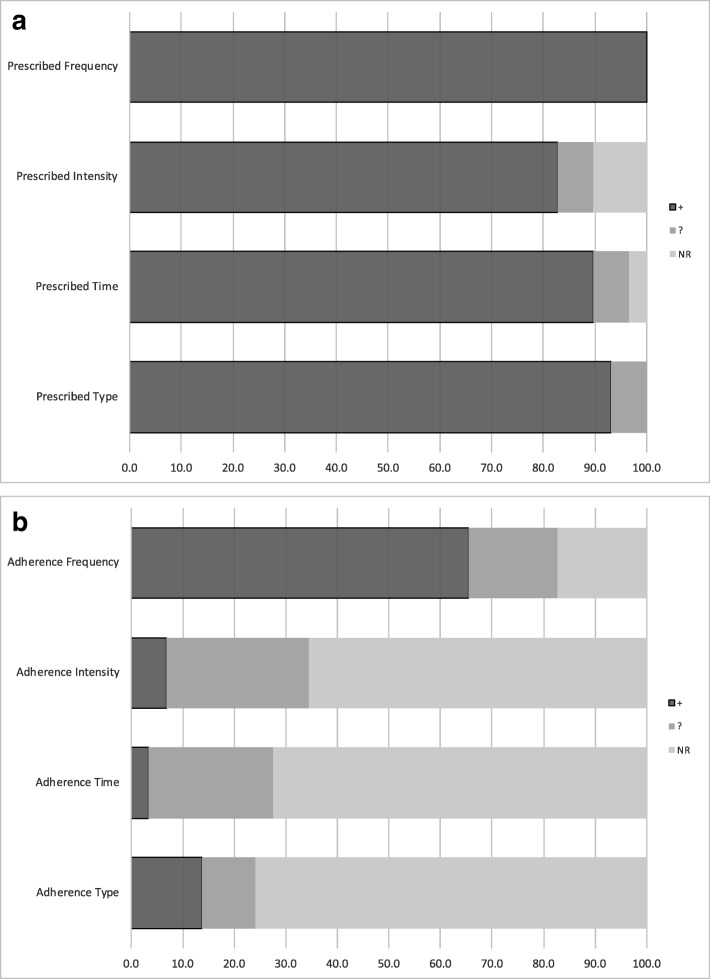
**a** Reporting of exercise prescription in study methods. **b** Reporting of adherence to exercise prescription in study results

Reporting of each component of the exercise prescription are displayed in Fig. 3a. Reporting of the components of the exercise prescription was generally high, with five (71%) aerobic [22, 25–28], all five (100%) resistance [23, 31–34], seven (70%) combined [19, 21, 35–39] and six (86%) multi-arm trials [24, 42, 43] adequately reporting all four components of the exercise prescription. Prescribed intensity was unclear in one (10%) combined [41] and one (14%) multi-arm trial [43] and was not at all reported in two (29%) aerobic [29, 30] and one (10%) combined trial [20]. Prescribed duration of exercise was unclear in one (10%) combined [41] and one (14%) multi-arm trial [43], and was not reported in one (10%) combined study [20]. Prescribed type of exercise was unclear in two (20%) combined studies [40, 41].

### Reporting of adherence to the prescribed intervention

Reporting of adherence to the prescribed interventions, or actual exercise completed by participants is displayed in Fig. 3b. No studies adequately reported adherence to all four components of the prescribed exercise program. Two (29%) aerobic [22, 26], four (40%) combined [20, 36, 40, 41] and two (29%) multi-arm interventions [42] did not adequately report any component of adherence.

Frequency of exercise completed was the most commonly reported component, with three (43%) aerobic [27, 28, 30], all five (100%) resistance [23, 31–34], six (60%) combined [19, 21, 35, 37–39] and five (71%) multi-arm trials [24, 43] adequately reporting frequency of exercise completed. One (14%) aerobic [29], two (20%) combined [36, 40], and two (29%) multi-arm trials [42] were unclear in their reporting of frequency of exercise completed, and three (43%) aerobic [22, 25, 26] and two (20%) combined studies [20, 41] did not report adherence to prescribed frequency at all.

Intensity of exercise completed was reported fully in only two (29%) aerobic studies [25, 27]. Adherence to intensity was unclear in three (43%) aerobic [26, 28, 30], two (40%) resistance [33, 34], and three (30%) combined studies [19, 35, 40]. Adherence to intensity prescribed was not at all described in the remaining two (29%) aerobic [22, 29], three (60%) resistance [23, 31, 32], seven (70%) combined [20, 21, 36–39, 41] and all seven (100%) multi-arm trials [24, 42, 43].

Duration of exercise completed was only reported in one (14%) aerobic trial [25]. Duration was unclear in three (43%) aerobic [26–28], one (20%) resistance [34], and three (30%) combined studies [19, 39, 40]. Duration was not at all reported in the remaining three (43%) of aerobic [22, 29, 30], four (80%) of resistance [23, 31–33], seven (70%) combined [20, 21, 35–38, 41], and all seven (100%) multi-arm trials [24, 42, 43].

The type of exercise completed was fully reported by four (57%) aerobic studies [25, 28–30]. Adherence to exercise type was unclear in one (14%) aerobic [27], one (20%) resistance [34], and one (10%) combined study [40], and was not at all reported in the remaining two (29%) aerobic [22, 26], four (80%) resistance [23, 31–33], 9 (90%) combined [19–21, 35–39, 41] and all seven (100%) multi-arm trials [24, 42, 43].

## Discussion

The papers included in this review of exercise programming and compliance in RCTs in men diagnosed with prostate cancer include data from 1891 men receiving various types of treatment for prostate cancer currently or in the past. The efficacy of exercise reported varied across trials and outcomes, but was generally positive; of the twenty papers that specified a primary outcome, 75% reported statistically significant changes in that outcome, and all but two trials found significant changes in at least one secondary outcomes.

Consistent with our previous findings in women with breast cancer, reporting of adherence to the exercise prescription was poor. Without greater information on compliance to the exercise prescription, it remains unclear what exercise dose is actually needed to achieve desired outcomes mirroring those observed in the studies presented.

No study reported all components, and three studies failed to report any indication of adherence. Frequency of exercise performed was most commonly reported, usually as a percentage of sessions attended in a supervised program or number of exercise sessions per week in a home-based intervention. This is not surprising given the relatively straightforward methods needed to collect this data. Reporting adherence to prescribed intensity may be more logistically challenging but is nonetheless important for replication and implementation. In an aerobic exercise intervention for example, average heart rate during an exercise session would require use of a heart rate monitor. Other methods, such as rating of perceived exertion (RPE) could be used if objective monitoring is not possible due to budgetary or logistic constraints. In addition to the actual FITT adherence, modifications needed to the exercise prescription, or the number of participants requiring substantial modifications would be interesting and important data to report.

As found in our previous reviews of breast and all other cancer types, attention to the standard principles of exercise training was not strong across the literature reviewed. No studies included in this review appropriately applied all principles of exercise training, and only nine studies appropriately reported more than half (i.e., four or five of the six) principles. Lack of attention to principles of exercise training may result in an underestimation of the true benefit that exercise may have at a larger population level. From a practice standpoint, inattention to training principles leaves the fitness professional without the information s/he may need to prescribe a training program for an individual client. When the exercise prescribed does not match the desired outcomes or inappropriate populations are selected (lack of attention to specificity), failure to see improvements in the primary outcome of interest may be due to the prescription chosen rather than lack of efficacy of exercise itself. In the papers included, we saw a substantial difference in attention to specificity between aerobic only (43%) and resistance only (100%) interventions. Body composition, and bone mineral density are key concerns for men with prostate cancer, particularly those on androgen deprivation therapy, and are a common target for exercise interventions. It is well known that weight bearing exercise, specifically moderate-high intensity resistance training is required to elicit changes in bone mineral density in healthy populations [44]. A popular type of exercise that is known to reduce fatigue during prostate cancer treatment is walking, but this modality would be ineffective if the goal were to prevent ADT-induced bone loss. Thus interventions who chose aerobic exercise only for this purpose were assigned a NR for this principle. In this review, specificity was appropriately applied most commonly, and is a more easily determined component of the prescription and should be continued in future trials and in translation to practice.

The principle of progression considers that as the body adapts over time to the training stimulus, in order to see clinically meaningful improvements, and continued improvement and maintenance of outcomes over time the training volume (intensity, frequency and duration) must continue to be altered over time [45]. In this review, just over half of studies included adequately reported progressive exercise prescriptions throughout the intervention period. Progression, and specifically the rate of progression of the exercise prescription is also important to note from a safety perspective. This information is critical for exercise professionals, as translation of exercise into community- or clinical- settings should be based on the RCT evidence accumulated to date. Progression that occurs too quickly could result in increased risk of injury, and too slowly could reduce efficacy, and lead to frustration and ultimately lack of compliance if individuals fail to see results. Similar to our previous reviews, we note that resistance training interventions are typically better at reporting progression in their prescription than aerobic interventions. This may be due to the perceived ease of instruction and recording of increased resistance weight. Although there is a reasonable upper limit to aerobic exercise frequency and duration that individuals will be able to complete from a logistics perspective, progression of intensity of exercise, through either heart rate target or RPE is important to elicit an adequate training stimulus.

Equally important considerations in ensuring that an adequate exercise stimulus or dose of exercise is delivered in order to expect improvements in a particular health outcome are the related principles of overload and initial values. Each of these principles were adequately reported by roughly half of included studies. To ensure participants experience gains in physical fitness, it is essential that exercise interventions be prescribed at a higher volume relative to measured baseline levels. Without knowing whether or not adequate overload was applied relative to an individual’s initial value of an outcome, participants may be prescribed less activity than reported at baseline, which introduces the potential for detraining during the intervention. On the other hand, a participant could receive a prescription that is too vigorous for them increasing the risk of adverse events or dropout. The population of men with prostate cancer represent a heterogeneous group; they tend to be older, have a variety of comorbidities, be physically inactive and receive treatments with side effects that accelerate age-related fitness declines. Given this, as well as individual differences in responses to exercise, prescribing exercise relative to a man’s starting capacity is critically important.

In this review, no study evaluated the principle of reversibility, and only 28% of studies examined the principle of diminishing returns. Understanding the trajectory of change over time in expected outcomes across an intervention is necessary to ensure that adequate overload is maintained by progressing the training program. For example, in resistance training interventions men will increase muscle strength over time such that a given weight represents less of their maximum capacity (e.g., 1-RM) and thus weakens the training stimulus. Knowing that diminishing returns is likely to occur, particularly in longer intervention periods, investigators and fitness professionals should build in periodic reassessments to recalibrate training volume. Measuring and reporting on these components may be logistically more challenging during an exercise intervention, as multiple time points of assessment are needed. When a full outcome assessment is not feasible, we urge investigators to consider the use of a shorter test battery or even proxy measures of the outcome of interest (example self-report body weight vs. in-person weigh in) at time points throughout the intervention to help us to better understand trajectory of change and adjust the exercise prescription as needed.

Reversibility is also a challenging principle to attend to, especially outside of the context of a supervised intervention. Reversibility of training effects may occur when a specific intervention ends and participants do not continue on their own, or due to lack of compliance during the intervention period. Understanding the long term effects on relevant outcomes after an intervention, particularly a supervised intervention, ends is important for the translation of research to practice. Knowledge of these aspects will help improve exercise prescription from an exercise physiology perspective, but may also inform incorporation of behaviour change techniques for long-term maintenance and sustainability.

One consideration that must be made when interpreting findings from this review is that study authors were not contacted for missing data. With respect to the attention to principles of exercise training, some of these studies may have carefully considered these in the design of their intervention but assigned a rating of unclear or NR in this review. We strongly urge all authors of exercise trials to carefully report all details of the intervention. Given that online appendices or supplementary materials are now common in most scientific journals, there is a greater opportunity to report these aspects in future studies while still adhering to strict word and page limits of the articles themselves. We also recommend authors follow the guidelines published in the recently published Consensus on Exercise Reporting Template (CERT), including the frequency, intensity, duration and type of exercise prescribed under section 13, “When, How Much” and exercise actually completed under section 16, “How Well: Planned, actual” [46].

## Conclusions

As the evidence base for the effect of exercise in men diagnosed with prostate cancer grows, it is important for researchers to apply the basic principles of exercise training in the design of their intervention, and to fully report all components of the exercise prescription (or dose) assigned and received, in order to continue to move the field of exercise oncology forward. This will allow better translation of evidence-based research into clinical practice and allow for greater fidelity of the anticipated response to a prescribed exercise program.

## Abbreviation

RCT: Randomized Controlled Trial

## References

[1] Canadian Cancer Society's Advisory Committee on Cancer Statistics: Canadian Cancer Statistics 2017. In*.* Toronto, ON: Canadian Cancer Society; 2017.

[2] Attard G, Parker C, Eeles RA, Schroder F, Tomlins SA, Tannock I, Drake CG, de Bono JS (2016). Prostate cancer. Lancet.

[3] Taylor LG, Canfield SE, Du XL (2009). Review of major adverse effects of androgen-deprivation therapy in men with prostate cancer. Cancer.

[4] Gardner JR, Livingston PM, Fraser SF (2014). Effects of exercise on treatment-related adverse effects for patients with prostate cancer receiving androgen-deprivation therapy: a systematic review. J Clin Oncol.

[5] Farris MS, Kopciuk KA, Courneya KS, McGregor SE, Wang Q, Friedenreich CM. Associations of postdiagnosis physical activity and change from prediagnosis physical activity with quality of life in prostate cancer survivors. Cancer Epidemiol Biomark Prev. 2017;26(2):179–87. 10.1158/1055-9965.EPI-16-0465 27677728

[6] Vashistha V, Singh B, Kaur S, Prokop LJ, Kaushik D (2016). The effects of exercise on fatigue, quality of life, and psychological function for men with prostate cancer: systematic review and meta-analyses. Eur Urol Focus.

[7] Keilani M, Hasenoehrl T, Baumann L, Ristl R, Schwarz M, Marhold M, Sedghi Komandj T, Crevenna R (2017). Effects of resistance exercise in prostate cancer patients: a meta-analysis. Support Care Cancer.

[8] Friedenreich CM, Wang Q, Neilson HK, Kopciuk KA, McGregor SE, Courneya KS (2016). Physical activity and survival after prostate cancer. Eur Urol.

[9] Campbell KL, Neil SE, Winters-Stone KM (2012). Review of exercise studies in breast cancer survivors: attention to principles of exercise training. Br J Sports Med.

[10] Winters-Stone KM, Neil SE, Campbell KL (2014). Attention to principles of exercise training: a review of exercise studies for survivors of cancers other than breast. Br J Sports Med.

[11] Hoffman J. Physiological Aspects of Sport Training and Performance. Champaign: Human Kinetics; 2002.

[12] Neil-Sztramko SE, Winters-Stone KM, Bland KA, Campbell KL (2019). Updated systematic review of exercise studies in breast cancer survivors: attention to the principles of exercise training. Br J Sports Med..

[13] Courneya KS, Jones LW, Peddle CJ, Sellar CM, Reiman T, Joy AA, Chua N, Tkachuk L, Mackey JR (2008). Effects of aerobic exercise training in anemic cancer patients receiving darbepoetin alfa: a randomized controlled trial. Oncologist.

[14] Courneya KS, Sellar CM, Stevinson C, McNeely ML, Peddle CJ, Friedenreich CM, Tankel K, Basi S, Chua N, Mazurek A (2009). Randomized controlled trial of the effects of aerobic exercise on physical functioning and quality of life in lymphoma patients. J Clin Oncol.

[15] Alberga AS, Segal RJ, Reid RD, Scott CG, Sigal RJ, Khandwala F, Jaffey J, Wells GA, Kenny GP (2012). Age and androgen-deprivation therapy on exercise outcomes in men with prostate cancer. Support Care Cancer.

[16] Bourke L, Gilbert S, Hooper R, Steed LA, Joshi M, Catto JW, Saxton JM, Rosario DJ (2014). Lifestyle changes for improving disease-specific quality of life in sedentary men on long-term androgen-deprivation therapy for advanced prostate cancer: a randomised controlled trial. Eur Urol.

[17] Buffart LM, Galvao DA, Chinapaw MJ, Brug J, Taaffe DR, Spry N, Joseph D, Newton RU (2014). Mediators of the resistance and aerobic exercise intervention effect on physical and general health in men undergoing androgen deprivation therapy for prostate cancer. Cancer.

[18] Gilbert SE, Tew GA, Fairhurst C, Bourke L, Saxton JM, Winter EM, Rosario DJ (2016). Effects of a lifestyle intervention on endothelial function in men on long-term androgen deprivation therapy for prostate cancer. Br J Cancer.

[19] Bourke L, Doll H, Crank H, Daley A, Rosario D, Saxton JM (2011). Lifestyle intervention in men with advanced prostate cancer receiving androgen suppression therapy: a feasibility study. Cancer Epidemiol Biomark Prev.

[20] Culos-Reed SN, Robinson JW, Lau H, Stephenson L, Keats M, Norris S, Kline G, Faris P (2010). Physical activity for men receiving androgen deprivation therapy for prostate cancer: benefits from a 16-week intervention. Support Care Cancer.

[21] Galvao DA, Taaffe DR, Spry N, Joseph D, Newton RU (2010). Combined resistance and aerobic exercise program reverses muscle loss in men undergoing androgen suppression therapy for prostate cancer without bone metastases: a randomized controlled trial. J Clin Oncol.

[22] Monga U, Garber SL, Thornby J, Vallbona C, Kerrigan AJ, Monga TN, Zimmermann KP (2007). Exercise prevents fatigue and improves quality of life in prostate cancer patients undergoing radiotherapy. Arch Phys Med Rehabil.

[23] Segal RJ, Reid RD, Courneya KS, Malone SC, Parliament MB, Scott CG, Venner PM, Quinney HA, Jones LW, D'Angelo ME (2003). Resistance exercise in men receiving androgen deprivation therapy for prostate cancer. J Clin Oncol.

[24] Segal RJ, Reid RD, Courneya KS, Sigal RJ, Kenny GP, Prud'Homme DG, Malone SC, Wells GA, Scott CG, Slovinec D'Angelo ME (2009). Randomized controlled trial of resistance or aerobic exercise in men receiving radiation therapy for prostate cancer. J Clin Oncol.

[25] Windsor PM, Nicol KF, Potter J (2004). A randomized, controlled trial of aerobic exercise for treatment-related fatigue in men receiving radical external beam radiotherapy for localized prostate carcinoma. Cancer.

[26] Eriksen AK, Hansen RD, Borre M, Larsen RG, Jensen JM, Overgaard K, Borre M, Kyro C, Landberg R, Olsen A (2017). A lifestyle intervention among elderly men on active surveillance for non-aggressive prostate cancer: a randomised feasibility study with whole-grain rye and exercise. Trials.

[27] Hvid T, Lindegaard B, Winding K, Iversen P, Brasso K, Solomon TP, Pedersen BK, Hojman P (2016). Effect of a 2-year home-based endurance training intervention on physiological function and PSA doubling time in prostate cancer patients. Cancer Causes Control.

[28] Jones LW, Hornsby WE, Freedland SJ, Lane A, West MJ, Moul JW, Ferrandino MN, Allen JD, Kenjale AA, Thomas SM (2014). Effects of nonlinear aerobic training on erectile dysfunction and cardiovascular function following radical prostatectomy for clinically localized prostate cancer. Eur Urol.

[29] Pernar CH, Fall K, Rider JR, Markt SC, Adami HO, Andersson SO, Valdimarsdottir U, Andren O, Mucci LA (2017). A walking intervention among men with prostate cancer: a pilot study. Clin Genitourin Cancer.

[30] Uth J, Hornstrup T, Schmidt JF, Christensen JF, Frandsen C, Christensen KB, Helge EW, Brasso K, Rorth M, Midtgaard J (2014). Football training improves lean body mass in men with prostate cancer undergoing androgen deprivation therapy. Scand J Med Sci Sports.

[31] Nilsen TS, Raastad T, Skovlund E, Courneya KS, Langberg CW, Lilleby W, Fossa SD, Thorsen L (2015). Effects of strength training on body composition, physical functioning, and quality of life in prostate cancer patients during androgen deprivation therapy. Acta Oncol.

[32] Norris MK, Bell GJ, North S, Courneya KS (2015). Effects of resistance training frequency on physical functioning and quality of life in prostate cancer survivors: a pilot randomized controlled trial. Prostate Cancer Prostatic Dis.

[33] Winters-Stone KM, Dobek JC, Bennett JA, Maddalozzo GF, Ryan CW, Beer TM (2014). Skeletal response to resistance and impact training in prostate cancer survivors. Med Sci Sports Exerc.

[34] Winters-Stone KM, Lyons KS, Dobek J, Dieckmann NF, Bennett JA, Nail L, Beer TM (2016). Benefits of partnered strength training for prostate cancer survivors and spouses: results from a randomized controlled trial of the exercising together project. J Cancer Surviv.

[35] Cormie P, Galvao DA, Spry N, Joseph D, Chee R, Taaffe DR, Chambers SK, Newton RU (2015). Can supervised exercise prevent treatment toxicity in patients with prostate cancer initiating androgen-deprivation therapy: a randomised controlled trial. BJU Int.

[36] Galvao DA, Taaffe DR, Cormie P, Spry N, Joseph DJ, Chambers SK, Gardiner RA, Bolam K, Wall BA, Newton RU. A multicenter yearlong randomized controlled trial of different exercise modalities in prostate cancer survivors on androgen deprivation therapy. J Clin Oncol. 2014;32(15).

[37] Gaskin CJ, Craike M, Mohebbi M, Courneya KS, Livingston PM (2017). A clinician referral and 12-week exercise training program for men with prostate cancer: outcomes to 12 months of the ENGAGE cluster randomized controlled trial. J Phys Act Health.

[38] Hojan K, Kwiatkowska-Borowczyk E, Leporowska E, Gorecki M, Ozga-Majchrzak O, Milecki T, Milecki P (2016). Physical exercise for functional capacity, blood immune function, fatigue, and quality of life in high-risk prostate cancer patients during radiotherapy: a prospective, randomized clinical study. Eur J Phys Rehabil Med.

[39] Hojan K, Kwiatkowska-Borowczyk E, Leporowska E, Milecki P (2017). Inflammation, cardiometabolic markers, and functional changes in men with prostate cancer. A randomized controlled trial of a 12month exercise program. Pol Arch Intern Med.

[40] Kim SH, Seong DH, Yoon SM, Choi YD, Choi E, Song Y, Song H (2018). The effect on bone outcomes of home-based exercise intervention for prostate cancer survivors receiving androgen deprivation therapy: a pilot randomized controlled trial. Cancer Nurs.

[41] Sajid S, Dale W, Mustian K, Kotwal A, Heckler C, Porto M, Fung C, Mohile SG (2016). Novel physical activity interventions for older patients with prostate cancer on hormone therapy: a pilot randomized study. J Geriatr Oncol.

[42] Santa Mina D, Alibhai SM, Matthew AG, Guglietti CL, Pirbaglou M, Trachtenberg J, Ritvo P (2013). A randomized trial of aerobic versus resistance exercise in prostate cancer survivors. J Aging Phys Act.

[43] Wall BA, DA GA, Fatehee N, Taaffe DR, Spry N, Joseph D, Hebert JJ, Newton RU: Exercise improves VO2max and body composition in androgen deprivation therapy-treated prostate cancer patients. Med Sci Sports Exerc. 2017;49(8):1503–10. 10.1249/MSS.0000000000001277 28319589

[44] Kohrt WM, Bloomfield SA, Little KD, Nelson ME, Yingling VR (2004). American College of Sports M: American College of Sports Medicine position stand: physical activity and bone health. Med Sci Sports Exerc.

[45] Garber CE, Blissmer B, Deschenes MR, Franklin BA, Lamonte MJ, Lee IM, Nieman DC, Swain DP (2011). American College of Sports M: American College of Sports Medicine position stand. Quantity and quality of exercise for developing and maintaining cardiorespiratory, musculoskeletal, and neuromotor fitness in apparently healthy adults: guidance for prescribing exercise. Med Sci Sports Exerc.

[46] Slade SC, Dionne CE, Underwood M, Buchbinder R (2016). Consensus on exercise reporting template (CERT): explanation and elaboration statement. Br J Sports Med..

